# Attenuation correction for PET/MRI to measure tracer activity surrounding total knee arthroplasty

**DOI:** 10.1186/s41824-022-00152-3

**Published:** 2022-11-07

**Authors:** Caleigh E. Bourdon, Zachary J. Koudys, Brent A. Lanting, C. Thomas Appleton, Jonathan D. Thiessen, Matthew G. Teeter

**Affiliations:** 1 Department of Medical Biophysics, Schulich School of Medicine & Dentistry, Western University, London, Canada; 2 Robarts Research Institute, London, Canada; 3 Lawson Health Research Institute, London, Canada; 4 Department of Surgery, Schulich School of Medicine & Dentistry, Western University, London, Canada; 5 Department of Medicine, Schulich School of Medicine & Dentistry, Western University, London, Canada; 6 Department of Medical Imaging, Schulich School of Medicine & Dentistry, Western University, London, Canada

**Keywords:** Hybrid PET/MRI, PET quantification, Total knee replacement, Metal artifacts, MR-based attenuation correction, Inpainting

## Abstract

**Background:**

Positron emission tomography (PET) in combination with magnetic resonance imaging (MRI) could allow inflammatory complications near total knee arthroplasty (TKA) to be studied early in their development. However, attenuation of the PET signal by the metal TKA implants imparts substantial error into measurements of tracer activity, and conventional MR-based attenuation correction (AC) methods have large signal voids in the vicinity of metal implants.

**Purpose:**

To evaluate a segmentation-based AC approach to measure tracer uptake from PET/MRI scans near TKA implants.

**Methods:**

A TKA implant (Triathlon, Stryker, Mahwah, USA) was implanted into a cadaver. Four vials were filled with [^18^F]fluorodeoxyglucose with known activity concentration (4.68 MBq total, 0.76 MBq/mL) and inserted into the knee. Images of the knee were acquired using a 3T PET/MRI system (Biograph mMR, Siemens Healthcare, Erlangen, Germany). Models of the implant components were registered to the MR data using rigid-body transformations and the other tissue classes were manually segmented. These segments were used to create the segmentation-based map and complete the AC. Percentage error of the resulting measured activities was calculated by comparing the measured and known amounts of activity in each vial.

**Results:**

The original AC resulted in a percentage error of 64.1% from the known total activity. Errors in the individual vial activities ranged from 40.2 to 82.7%. Using the new segmentation-based AC, the percentage error of the total activity decreased to 3.55%. Errors in the individual vials were less than 15%.

**Conclusions:**

The segmentation-based AC technique dramatically reduced the error in activity measurements that result from PET signal attenuation by the metal TKA implant. This approach may be useful to enhance the reliability of PET/MRI measurements for numerous applications.

## Background

Total knee arthroplasty (TKA) is a surgical procedure involving the removal of a large portion of the knee joint and replacing it with a metallic implant. This surgery is commonly used to treat severe osteoarthritis (Dieppe et al. 1999). Although TKA is often successful in reducing pain and improving the patient’s knee’s mobility, some patients suffer from complications (Dieppe et al. 1999; Granchi et al. 2008). Complications and modes of failure following TKA frequently involve inflammation within joint tissues, including arthrofibrosis, reactions to wear debris causing osteolysis and aseptic loosening, and periprosthetic infection (Granchi et al. 2008; Healy et al. 2013).

Typically, these inflammatory issues are not recognized until they are at advanced stages when morphological changes become apparent on conventional imaging modalities such as radiography, computed tomography (CT), and magnetic resonance imaging (MRI) (Li et al. 2016; Tu et al. 2018; Bruggen et al. 2018). Late-stage identification of complications makes their treatment challenging and often revision surgery is required; this involves the removal of the primary implant along with any fibrotic tissue and the insertion of a new implant (Moya-Angeler et al. 2017). Although they are frequently performed to treat primary TKA (pTKA) complications, revision TKA (rTKA) surgeries are not always effective and have increased morbidity and operative costs when compared to pTKA surgeries (Cheuy et al. 2017).

Positron emission tomography (PET) utilizes tracers that interact with biological molecules, such as proteins, enabling cellular metabolic activity to be measured, potentially allowing inflammatory processes to be identified and studied much earlier in their development using tracers such as [^18^F]FEPPA or [^18^F]FDG (Korbin et al. 2020; Vignal et al. 2018). This could improve the possibility of successful treatment or prevention of complications without implant revision. Although most orthopedic applications have utilized PET/CT, PET/MRI has advantages including better soft tissue contrast and less ionizing radiation (Koob et al. 2019; Ehman et al. 2017). PET/MR could be used to evaluate tracer activity in the joints of TKA recipients could lead to greater understanding of pathological inflammation leading to implant failure and be a non-invasive tool to evaluate new therapies to avoid the need for revision surgeries. However, attenuation of the PET signal by the metal TKA implants imparts substantial error into measurements of tracer activity. With PET/CT, attenuation correction (AC) can be performed from the CT data, albeit with CT metal artifacts due to beam hardening and scatter (Ehman et al. 2017). MRI cannot directly measure tissue density, and metal artifacts, such as signal voids, interfere with the generation of a linear attenuation coefficient (LAC) map, or µ map, leading to inaccuracies in the measured PET data (Chen and An 2017; Wagenknecht et al. 2013). Current AC approaches for PET/MRI images involve the use of dedicated metal artifact reduction sequences and post processing of MR-based µ maps (Wagenknecht et al. 2013). Slice encoding for metal artifact correction (SEMAC) and multi-acquisition variable-resonance image combination SeLective (MAVRIC-SL) are dedicated metal artifact reduction sequences that employ view angle tilting and additional slice encoding to correct for metal-induced signal distortions (Lu et al. 2009; Koch et al. 2011; Cho et al. 1988). Another method for the AC of PET/MRI images involves inpainting, a technique which fills the signal voids caused by metal implants in the µ map with an LAC value, such as that of soft tissue, in the µ map (Ladefoged et al. 2013). Ladefoged et al. (2013) and Schramm et al. (2014) describe a foundational approach to DIXON based µ map correction for AC in PET/MRI which involve inpainting with the LAC of soft tissue and have shown to improve the accuracy of the resulting PET signal (;). Other methods, such as automatic or semiautomatic algorithms, atlas-based methods and deep learning neural networks, have been proposed for AC (Burger et al. 2015; Arabi and Zaidi 2022, 2020; Schramm and Ladefoged 2019). Atlas-based methods have been proposed that deform atlas datasets on to the target MR dataset to calculate the most probable tissue and bone segmentation in the region of metal artifact induced signal loss. This method showed improvements over traditional manual inpainting but was not as accurate as deep learning methods (Arabi and Zaidi 2022). Arabi and Zaidi showed that a deep learning-based approach could extract tissue data surrounding the metal artifact in MR images and be used to automatically fill the signal void created by metal implants. However, the implants were mainly dental fillings that are much smaller than orthopedic prosthesis and more data are needed on the viability of this approach in large endoprosthesis (Arabi and Zaidi 2020). Fuin et al. (2017) discussed a novel algorithmic method for estimating the position, size, and LAC of metal endoprosthesis. A maximization algorithm was used on whole body PET data to define metal implant boundaries and LAC values and showed improvements over traditional CT-based LAC calculations. This method relies on assumptions that implant components have no radioactivity and that the PET tracer has good spatial distribution, which may not be true in all scenarios.

The novel method for AC proposed in this study combines previously discussed methods of µ map inpainting with manual placement of implant CAD models and may be useful for accurate µ map reconstruction in knee replacements when implant models are available. Information about the size, shape, and density of the implant components are often available through joint prosthesis vendors and may provide unparalleled accuracy in PET/MRI AC around knee endoprosthesis. Therefore, the purpose of this study was to evaluate a segmentation-based inpainting AC approach that incorporates information from available implant CAD models to accurately measure tracer uptake from PET/MRI scans near TKA implants.

## Methods

A cruciate retaining TKA implant (Triathlon, Stryker, Mahwah, USA) consisting of a cobalt-chromium femoral and tibial component, and a polyethylene tibial insert was implanted into a cadaver knee by an orthopedic surgeon (BAL). Four vials were filled with a known activity concentration of [^18^F]fluorodeoxyglucose (FDG) (4.68 MBq total, 0.76 MBq/mL). These vial volumes and activities are shown in Table 1.

**Table 1:** The known total volume of four vials containing [^18^F]FDG

Known vial volume and activities (gold standard)					
Vial	Reference	Anterior	Mid	Posterior	Total
Tracer volume (mL)	1.52	1.53	1.56	1.57	6.18
Activity (MBq)	1.15	1.16	1.18	1.19	4.68

Vials were placed in the anterior, mid joint, and posterior positions relative to the knee joint. A reference vial was positioned far from implant components and on the surface of the skin. The radioactivity contained in each vial was reported

The vials were inserted into the knee: one posteriorly within the joint, one between the femoral and tibial components, one anteriorly within the joint, and one on the surface of the skin, to act as a reference away from the metallic components.

**Fig. 1:**
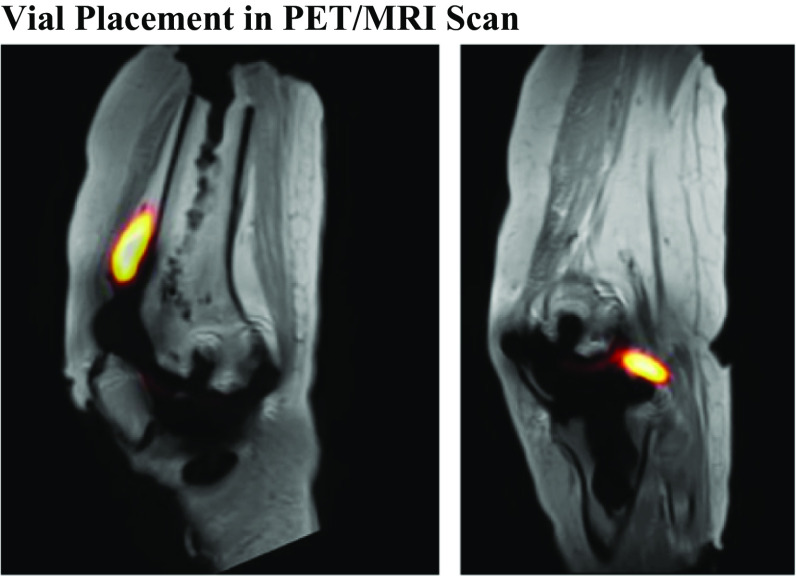
Sagittal views of fused PET/MRI scans of the cadaver knee demonstrating vial placement in the anterior (left) and mid (right) joint

Sagittal and coronal T_1_-weighted images of the knee (Fig. 1) were acquired on a 3 T PET/MRI system (Biograph mMR, Siemens Healthcare, Erlangen, Germany) using a high bandwidth fast spin-echo (FSE) sequence with metal artifact reduction techniques (Siemens *Syngo* Advanced WARP), including view angle tilting (VAT) for in-plane distortion correction and SEMAC for the correction of through-plane distortions. FSE pulse sequence parameters were; echo time = 5.4 ms, repetition time = 2910 ms, flip angle = 120°, bandwidth = 610 Hz/pixel, field-of-view = 320 mm, matrix size = 256 × 256, in-plane resolution = 1.25 mm, slice thickness = 3 mm, number of slices = 41 (sagittal) and 37 (coronal), VAT = 100%, and number of SEMAC phase-encoding steps = 15. A standard attenuation correction map based on the 2-point Dixon gradient echo pulse sequence was also acquired. All images were acquired using the mMR spine and body RF arrays and reconstructed with pre-scan normalization and 2D distortion correction. List-mode PET data was acquired simultaneously for 60 min. Attenuation-corrected PET images were reconstructed using a 3D ordered subset expectation maximization (OSEM) algorithm with 21 subsets and 3 iterations. Reconstructed PET images consisted of 127 slices with a matrix size = 344 × 344, zoom factor = 2, and a 2 mm Gaussian filter, resulting in an in-plane image resolution of (1.04 × 1.04) mm^2^ and slice thickness of 2.03 mm for the reconstructed PET images.

Three-dimensional CAD models of the implant components were manually registered to the MR data using an image computing platform (3D Slicer 4.10.2, slicer.org). Even with the use of the SEMAC sequence, the signal void caused by the metal implants extended slightly beyond the boundaries of the prosthesis. To reduce placement ambiguity, small landmarks, such as femoral anchor pins, were used during the registration. The other tissue classes (i.e., bone and soft tissue) were manually segmented based on the MR images. These segments were used as a mask over the original 2-point Dixon AC map generated by the PET/MRI system, which is susceptible to metal artifacts and has a significant signal void. With the exception of the bone segmentation, the areas of the map which were not affected by the metal-induced signal void were left unmodified.

Then, the respective LAC values of each material (Table 2) for 511 keV positron annihilation photons were added onto the map in place of the signal void. Most values were determined from the literature, and the value for the polyethylene was determined from a CT scan of the polyethylene spacer.

**Table 2:** LAC values used to construct the segmentation-based µ map

LAC values of tissue and implant components						
Material	Air (Wagenknecht et al 2013)	Soft tissue (Berker et al. 2012)	Adipose tissue (Berker et al. 2012)	Bone (Keereman et al. 2011)	Polyethylene	Cobalt-Chromium (Fuin et al. 2017)
LAC (cm^−1^)	0	0.0927	0.0854	0.13	0.079	0.72

Values were derived from literature or CT-based density calculation

This method was used to generate three different MR-based µ maps: (1) a simple map, with the signal void around the implant filled only with the LAC of soft tissue, (2) a map using the CAD models for a different system (Genesis II, Smith & Nephew, Memphis, TN) to represent an alternative implant model, and (3) the most accurate map, using the CAD models for the implant components matching those of the cadaver. One distinguishing factor of the alternative model is that it has a posterior stabilized design, while the cadaver’s model is cruciate retaining.

**Fig. 2:**
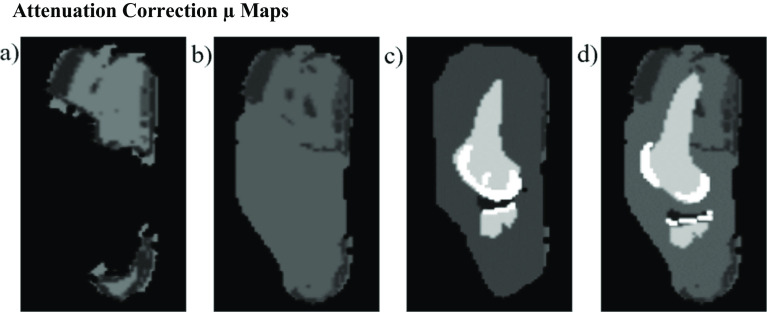
Attenuation correction µ maps generated in four ways of varying complexity. **a** Single slice of the original 2-point Dixon, **b** simple tissue, **c** alternative model, and **d** accurate model µ maps

A single slice of these maps is shown in Fig. 2. Each of these maps had the same LAC values for air, soft tissue, and bone. The maps which involved information from the CAD models had the same LAC value for the polyethylene component. Consistent with the manufacturer specifications in the accurate implant design, the femoral and tibial components in the µ map were filled with the LAC of cobalt-chromium. In the alternative implant map, the femoral and tibial component segments were also filled with the LAC of cobalt-chromium. These three AC maps were then used in the reconstruction of the original PET data. The time required to produce and analyze these µ maps was approximately 30–60 min.

Segmentations with the same shape and volume as each vial were used to measure activity concentration and total activity data. The segments were drawn to match the shape and size of the conical vial that held each tracer. To create these vial congruent segmentations, the PET data for the reference vial was used as a guide to draw the segmentation. This segmentation volume was compared to the known tracer volume in each vial to confirm that the segmentation was the proper size. The mean activity concentration within the segmentation for each vial was used as the measured activity concentration, in MBq/mL. The mean activity concentration multiplied by the segmentation volume was used to measure the total activity in each vial, in MBq. Additionally, the total activity in the cadaver was calculated without the use of segmentation. This measurement was not vial specific and encompassed the entire cadaver.

In a previous experiment, PET recovery coefficients (RC) were measured using a modified NEMA IEC Body Phantom containing acrylic cylinders with inner diameters ranging from 5 to 30 mm filled with a known activity concentration (Dassanayake et al. 2022). The RC curve was fit to an equation describing RC as a function of diameter (Croteau et al. 2010). The RC was determined to be 0.5167 and applied to the measured mean concentration values of each vial, based on a vial diameter of 10 mm. The RC correction accounts for the partial volume effect in PET, which affects quantitative PET analysis in small volumes of activity (Srinivas et al. 2009).

Percentage errors of the measured activities from the gold standard for the original, unmodified map as well as the three segmentation-based maps were calculated by comparing the measured tracer activity levels to the known tracer activities, the gold standard. Additionally, percentage errors of the measured activities compared to the reference were calculated by comparing the individual activities of the three vials internal to the joint to the reference vial on the skin outside of the joint. These errors were computed using percent error calculations (% Error = (Measured Activity-Gold Standard or Reference Activity)/(Gold Standard or Reference Activity) × 100). Completing comparisons to the gold standard as well as to the reference vial provide good representation of the range of error expected when quantifying activity in vivo, given that there are inherent errors in dose calibration and partial volume correction.

The creation of the accurate model segmentation-based µ map and the measurement of the resulting activities was completed a second time by a different observer. This allowed examination of the repeatability of the process and the inter-observer error related to the analysis.

## Results

The resulting percent errors from the gold standard for the total activity in the cadaver are shown in Table 3, the individual vial errors compared to the gold standard are shown in Table 4, and the individual vial errors compared to the reference are shown in Table 5.

**Table 3:** Percent errors of the total cadaver activity compared to the gold standard

Total percent error versus standard				
Map	Original	Simple tissue	Alternative model	Accurate model
Percent error	− 64.15%	− 26.51%	14.56%	3.55%

**Table 4:** Percent errors of the individual total vial activities and activity concentrations compared to the gold standard

Vial percent error versus standard								
Map	Original		Simple tissue		Alternative model		Accurate model	
	Total activity (%)	Activity concentration (%)	Total Activity (%)	Activity concentration (%)	Total activity (%)	Activity concentration (%)	Total activity (%)	Activity concentration (%)
Reference vial	− 41.2	− 40.2	− 11.3	− 9.62	2.59	4.56	− 6.99	− 5.20
Anterior vial	− 59.0	− 59.0	− 19.6	− 18.5	− 12.3	− 11.17	− 6.26	− 5.05
Middle vial	− 83.3	− 83.0	− 44.1	− 42.9	2.33	4.49	− 16.8	− 15.0
Posterior vial	− 66.8	− 66.2	− 42.8	− 40.3	− 1.66	2.63	− 5.17	− 1.02

**Table 5:** Percent errors of the individual total vial activities and activity concentrations compared to the reference vial

Total percent error versus reference								
Map	Original		Simple tissue		Alternative model		Accurate model	
	Total activity (%)	Activity concentration (%)	Total activity (%)	Activity concentration (%)	Total activity (%)	Activity concentration (%)	Total activity (%)	Activity concentration (%)
Anterior vial	− 29.7	− 31.4	− 8.66	− 9.86	− 13.9	− 15.05	1.50	0.164
Middle vial	− 70.9	− 71.5	− 35.4	− 36.9	2.31	0.066	− 8.23	− 10.4
Posterior vial	− 71.5	− 71.1	− 33.2	− 34.0	− 0.675	− 1.84	5.65	4.41

Table 6 shows the errors that were observed by the second observer when repeating the measurement on the accurate model.

**Table 6:** PET errors for total activity and activity concentration measured by the second observer using the accurate model µ map

Inter-observer reliability of accurate model		
	Accurate model	
	Total activity (%)	Activity concentration (%)
Reference vial	3.10	3.23
Anterior vial	− 1.35	− 1.79
Middle vial	− 6.83	− 4.18
Posterior vial	− 5.67	− 1.56

## Discussion

With the use of implant CAD models*,* this segmentation-based AC technique dramatically reduced the error in activity measurements that result from PET signal attenuation by the metal components of the TKA implant. An effect of proximity to the metal components on error was noted and should be accounted for when interpreting PET data near TKA implants; vials which were more surround by metal components were more impacted by signal attenuation and required more correction, which introduced more opportunity for error in CAD model placement. Additionally, any error in CAD model placement would be compounded in the middle vial which was surrounded on all sides by this increased source of error. This approach may be useful to enhance the reliability of PET/MRI measurements of cellular activity within knee joints following TKA.

It should also be noted that results using the alternative CAD model and accurate CAD model segmentation were similar, however the errors using the simple tissue segmentation were substantially greater. This suggests that replacing the signal void in the µ map only with the LAC of soft tissue is not sufficient, and implant CAD models should be used. Additionally, if the exact design of the implant is not known or are unavailable, an alternative model can be used with reasonable accuracy. AC was also completed using an alternative metal, Titanium, which resulted in significant errors when used in the femoral component of the implant. However, given that all patients are screened for metal implants prior to MRI for MRI safety reasons, and would have the metal implant type on their medical records, it is reasonable to assume that the metal type of an implant would be obtainable prior to PET/MR imaging and AC.

There were some variations in errors between each vial and when looking at the activity in the entire cadaver, even with the more accurate µ maps. The differences in errors between individual vials can be explained by their locations and proximities to the metal implant components. Additionally, when measuring the individual vial activities, any signal from outside the boundaries of the vials is not measured, while the measurement of the total phantom activity does count this additional activity. This explains why the error observed when looking at the total cadaver activity is positive, or is the result of over-estimation, while the individual vial measurements typically under-estimate, or have negative errors. Although a RC was used to correct for partial volume effects based on the vial diameter, there still exists some error in activity concentration estimates based on variability in region of interest selection and varying degrees of attenuation experienced by the individual vials based on their location in the knee. Additionally, there may be systemic errors in the estimates of the RCs (Dassanayake et al. 2022). Additionally, partial volume errors in objects approaching the intrinsic spatial resolution of the PET/MRI contribute to signals outside the boundaries of the vials, which are amplified by overlap of high-density components in the µ map and included in the total cadaver measurement. Thus, it is important to consider the location of the analyzed vials as well as the type of measurement when calculating the PET error. In the original and simple µ maps, there is an underestimation of density of the materials in the cadaver, leading to an under-correction and large negative error values.

Another inpainting MR-based AC method proposed by Ladefoged et al. (2013) showed a 48% increase in PET signal when the signal void was replaced with soft tissue. Schramm et al. (2014) also replaced signal voids with soft tissue using an automatic algorithmic protocol. This work reported a calculated PET SUV uptake improvement of 37–59% for whole body metal artifacts including endoprosthesis. A review article by Chen et al. evaluated the effectiveness of segmentation-based attenuation correction in whole body PET/MR of patients using four material classes, namely air, lung, soft tissue, and fat, and reported PET errors of 5–20% (Chen and An 2017). The present study was able to consider additional segmentation classes, such as bone, metal, and polyethylene, and resulted in an error reduction in calculated PET signal near metal endoprosthesis of 53.95–68% (Table 4). Therefore, the proposed method can be considered successful in reducing the error between the calculated PET signal before and after MR-based AC. As concluded by Schramm et al., manual inpainting methods will be necessary for the correction of PET images in the presence of large metal objects, until more sophisticated vendor solutions become available (Schramm and Ladefoged 2019).

In this study, the geometry and volume of each PET tracer vial were known which allowed the use of the RC. To translate this method to a clinical setting where the size and shape of ROIs are unknown prior to the scan, anatomical information from the MRI can be used to correct for partial volume errors.

Although some inter-observer error was observed, the mean difference in the PET errors calculated was 6.11% for the total activities and 5.49% for the activity concentration, with a maximum difference of 10.8% (percent error = 72.1%). Errors between observers can be explained by the fact that the implant model registration must be done manually, a process which is challenging, given the lasting artifacts in the MR images. The difference in inter-observer error was most significant in the middle vial with a total activity difference in error of 9.97% between observers. This may be due to this vial being the most surrounded by metal artifacts and most susceptible to error from improper CAD model placement. The development of an automation method would be useful for future applications to reduce inter-observer error.

This study had some limitations. First, as shown in a study by Teeter et al., the manufacturer-provided CAD models of the TKA implant components have been found to be slightly different from the actual implant components due to manufacturer variability (Teeter et al. 2009). This could have contributed to small inaccuracies in the segmentation-based µ map, which could have affected the final measured activities. Secondly, it is unlikely that the correct CAD models for every implant will be available at the time of imaging. Also, the registration of the implant components to the MR images was done manually, which was necessary due to the lasting artifacts present in the MR images, even with the use of the metal artifact reduction sequences. Next, manual registration is labor-intensive, making it difficult to apply in larger studies. A more simplified or automated method would be necessary for use in clinical studies with multiple patients. Finally, it is worth noting that the biograph mMR applies hardware attenuation corrections for the patient table and rigid spine RF array but not the flexible body RF array. This may contribute an additional 2–5% loss of total measured PET activity due to the presence of the flexible body RF array (Kartmann et al. 2013).

## Conclusion

This study introduced the use of implant CAD models for MR-based AC. When compared to a segmentation-based approach without the use of implant CAD models, the proposed method performed significantly better, whether the appropriate CAD model was used. Thus, this segmentation-based approach to MR-based AC was successful in improving the accuracy of PET/MR imaging around large metal implants.

## Data Availability

The datasets generated during and/or analyzed during the current study are available from the corresponding author on reasonable request.

## Abbreviations

TKA: Total knee arthroplasty
AC: Attenuation correction
CT: Computed tomography
MRI: Magnetic resonance imaging
pTKA: Primary total knee replacement
rTKA: Revision total knee replacement
PET: Positron emission tomography
LAC: Linear attenuation coefficient
SEMAC: Slice encoding for metal artifact correction
MAVRIC-SL: Multi-acquisition variable-resonance image combination selective
FDG: Fluorodeoxyglucose
FSE: Fast spin-echo
VAT: View angle tilt
OSEM: Ordered subset expectation maximization
RC: Recovery coefficient
